# Molecular Shield for Protection of Buckwheat Plants from UV-B Radiation

**DOI:** 10.3390/molecules27175577

**Published:** 2022-08-30

**Authors:** Ivan Kreft, Alena Vollmannová, Judita Lidiková, Janette Musilová, Mateja Germ, Aleksandra Golob, Blanka Vombergar, Darja Kocjan Ačko, Zlata Luthar

**Affiliations:** 1 Nutrition Institute, Tržaška 40, SI-1000 Ljubljana, Slovenia; 2 Biotechnical Faculty, University of Ljubljana, SI-1000 Ljubljana, Slovenia; 3 Faculty of Biotechnology and Food Sciences, Slovak University of Agriculture in Nitra, Tr. A. Hlinku 2, 949 76 Nitra, Slovakia; 4 The Education Centre Piramida Maribor, SI-2000 Maribor, Slovenia

**Keywords:** buckwheat, rutin, quercetin, flavonoid, UV radiation, altitude, climatic change

## Abstract

Tartary buckwheat (*Fagopyrum tataricum* (L.) Gaertn.) and common buckwheat (*Fagopyrum esculentum* Moench) are adapted to growing in harsh conditions of high altitudes. Ultraviolet radiation at high altitudes strongly impacts plant growth and development. Under the influence of ultraviolet radiation, protecting substances are synthesized in plants. The synthesis of UV-B defense metabolites is genetically conditioned, and their quantity depends on the intensity of the ultraviolet radiation to which the plants and plant parts are exposed. These substances include flavonoids, and especially rutin. Other substances with aromatic rings of six carbon atoms have a similar function, including fagopyrin, the metabolite specific for buckwheat. Defensive substances are formed in the leaves and flowers of common and Tartary buckwheat, up to about the same concentration in both species. In comparison, the concentration of rutin in the grain of Tartary buckwheat is much higher than in common buckwheat. Flavonoids also have other functions in plants so that they can protect them from pests and diseases. After crushing the grains, rutin is exposed to contact with the molecules of rutin-degrading enzymes. In an environment with the necessary humidity, rutin is turned into bitter quercetin under the action of rutin-degrading enzymes. This bitterness has a deterrent effect against pests. Moreover, flavonoids have important functions in human nutrition to prevent several chronic diseases, including obesity, cardiovascular diseases, gallstone formation, and hypertension.

## 1. Introduction

Tartary buckwheat (*Fagopyrum tataricum* (L.) Gaertn.) and common buckwheat (*Fagopyrum esculentum* Moench) originate on the mountain slopes of the Himalayas, and they grow in diverse situations. On the outskirts of the Himalayan mountains, both species continue to exist as wild plants [1,2,3]. The farmers collect the seeds from selected plants, reproduce them in the fields, and use the crop in their meals.

Tartary and common buckwheat are cultivated plants in the Himalayan mountains, and elsewhere in the world, mainly in China, Korea, Japan, India, Nepal, Bhutan, Pakistan, Kazakhstan, in Europe (Russia, Ukraine, Belarus, Latvia, Lithuania, Estonia, Sweden, Poland, Czech Republic, Italy, Slovakia, Slovenia, Bosnia and Herzegovina, Croatia, Hungary, Italy), Tanzania, South Africa, the United States, Canada, and Brazil [4].

Due to its robust husk and high content of protective phenolic substances, dormant Tartary-buckwheat seeds may remain alive in the soil for several years and, under favorable conditions, can be activated. Tartary buckwheat may appear as a weed in other crops and is often a weed in crops of common buckwheat. For the survival of Tartary-buckwheat plants, finding space, soil, and sunlight in competition with other plants is not the only important matter. These requirements are notable for plants that grow in places with more lush and dense vegetation. Tartary buckwheat survives on stony areas, at high altitudes, and under strong ultraviolet radiation. At even higher altitudes, the plants of Tartary buckwheat survived during evolution by gradually accumulating substances that allow this species to survive and reproduce on the less friendly stony heights of the Himalayas. Both the diversity and richness of Tartary-buckwheat substances protect the plants from unfriendly environmental effects, diseases, and grazing. However, these substances are also important in food to preserve human health.

Many new genes involved in rutin biosynthesis and regulation, aluminum-stress resistance, and drought- and cold-stress responses were identified in buckwheat [5]. The ability of buckwheat to tolerate high levels of abiotic stress is attributed to the emerging and expansion of several gene families involved in signal transduction, gene regulation, and membrane transport. These genetic resources have facilitated the discovery of physiologically and nutritionally relevant genes, and the possibility of the genetic improvement in the growth ability and nutritional value of Tartary buckwheat [5]. Researchers identified 769 single gene families to construct phylogenetic trees, which showed that the ancestors of Tartary buckwheat and sugar beet were located on a branch out of rosids and asterids, which further defines the divergence of Caryophyllales prior to the split of asterids and rosids [6]. The data indicate that the two species are distantly related to each other, which is consistent with their previous phylogenetic inclusion in different taxonomic families, as defined by the morphology.

**Figure 1:**
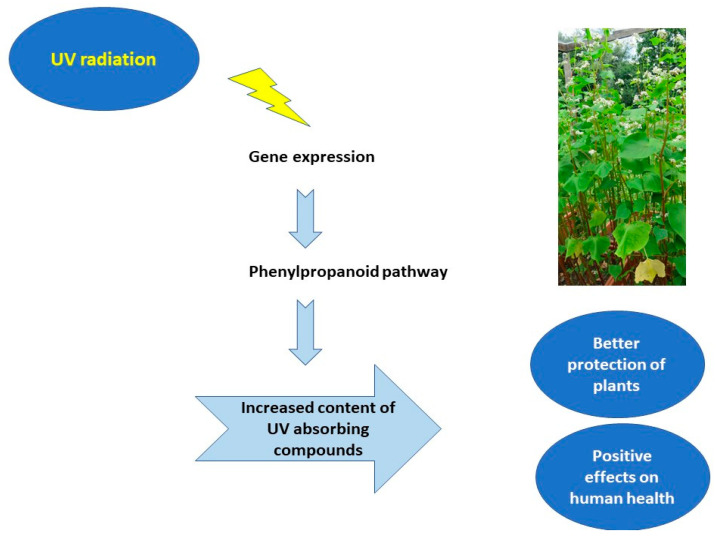
Pathway of impacts of UV-B radiation on buckwheat plants.

However, Zhang et al. [5] defined the changes in the Tartary buckwheat lineage that appeared after its divergence from its sugar-beet ancestors. These changes are obviously responsible for the adaptation of Tartary buckwheat to extremely adverse environmental conditions (Figure 1). They also demonstrated the importance of the reference genome by identifying genes included in the rutin biosynthetic pathway, and the myeloblastosis (MYB) transcription factors [5,7].

The mycorrhizal hyphae of buckwheat plants affect the growth and development of organisms in the soil [8,9]. By transferring various substances, including those that regulate plant growth, hyphae also transmit molecules that can influence the way organisms coexist in the soil. That mycorrhizal fungi that are found in common buckwheat were first discovered in Slovenia [8], and this was subsequently also confirmed in Tartary buckwheat [9]. The appearance of mycorrhizal fungi is, in buckwheat, connected with the content of flavonoid substances [9]. Buckwheat can activate some mineral elements in soil, including phosphorus [10,11]. Phosphorus is tightly attached to the mineral structure of the soil. The buckwheat roots have a special ability to activate phosphorus, making it available for plants and other organisms in the soil. The activation of phosphorus is very important for environmentally friendly farming.

In common buckwheat, its flowers form a nectar and attract insects, bringing genes with pollens from distant plants. Insects such as honeybees, wild bees, and butterflies are also pollinators. Producing food for insects, buckwheat contributes to the preservation of biodiversity in the environment. Common buckwheat flowers contain UV-reflecting substances, such as rutin, quercetin, and other phenolic substances; the insect-attracting effects of flavonoids in flowers has not yet been investigated in detail [12,13,14]. Research has shown that, near mixed forests with diverse flora and fauna, common buckwheat produces a higher yield than when plantation forests with monoculture forest trees neighbor the buckwheat fields, and it offers little opportunity for insect refuge [15].

The buckwheat grain is rich in nutrients, and particularly starch, proteins, and minerals. This treasure is protected by a hard pericarp that develops from the fruit’s wall.

## 2. Impact of Solar Radiation

The radiation emitted from the Sun to the Earth has a rich spectrum, from invisible UV radiation and visible light to thermal radiation.

Parts of visible light are very important for photosynthesis. Ultraviolet rays can damage the gentle tissue of plants [16]. Some plants have crystals or growths on the leaf surface that reflect, or at least disperse, harmful UV radiation [17]. Many plant species form substances that protect them from UV radiation.

**Figure 2:**
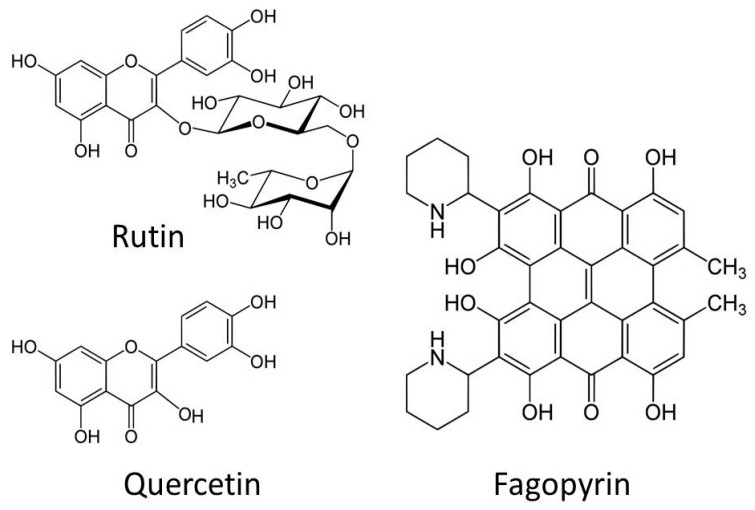
Molecular structures of rutin, quercetin, and fagopyrin.

These substances are mainly phenols, which are substances with aromatic rings made of six carbon atoms, with double ties and the carbon-atom-bound groups OH or others. Sometimes other molecules, such as sugars, are attached. In the case of buckwheat, among the particularly interesting are rutin, quercetin, and fagopyrin [18,19,20,21,22] (Figure 2).

These secondary metabolites with aromatic rings are built in buckwheat plants, some in common buckwheat, and significantly more in Tartary buckwheat. Genes and enzymes that allow the gradual buildup of phenolic substances have been studied primarily in Tartary buckwheat [7].

In the grain of common buckwheat, a small concentration of rutin was found (about 0.01%), while in the grain of Tartary buckwheat, we found it up to 1.4% [23,24]. There is also rutin in the leaves of both species, but the difference between the two types of plants is smaller than in the grain.

We tested the synthesis of rutin in buckwheat plants, which grew at different altitudes (about 300, 600, 800, and 1100 m). At an altitude of more than 1100 m on Javorje near Črna na Koroškem (north of Slovenia), more than 1.6% rutin was measured in the leaves of Tartary buckwheat, and in the leaves of common buckwheat, slightly less than 1.5% [25]. The highest concentration of rutin was present in the leaves of Tartary buckwheat, which grew at the highest altitude, where there is the strongest UV radiation. At all altitudes (about 300, 600, 800, and 1100 m), we also measured more rutin in the leaves of Tartary buckwheat than in the leaves of common buckwheat [26]. Rutin and also fagopyrin are UV-absorbing molecules, which is reflected in the increased content of both components in different parts and stages of plant development [27,28] (Table 1). 

Additional illumination of buckwheat with UV radiation increased the amount of rutin and other UV-absorbing substances [29]. Therefore, the ability of plants to synthesize defense substances is more pronounced in increased UV radiation loads. When plants are protected by a transparent film that retains most of the UV radiation, the synthesis of UV-absorbing substances is also reduced. The synthesis of these substances is energy demanding for the plant, and there are used assimilates for this. Thus, to some extent, the synthesis of protecting substances is avoided, if not needed, due to the radiation conditions. Even if there is no direct impact of UV rays, plants still form some protective substances, just in case they are suddenly exposed to strong solar radiation with UV rays, before they react with the increased activity of genes to produce enzymes that enable the synthesis of UV-absorbing substances. It is understandable that there are significantly fewer UV-absorbing substances in the roots compared with the leaves and flowers. The flowers are most exposed to solar radiation, and they contain the highest concentration of substances that protect them from UV radiation (Table 1).

In the Gaberščik et al. [30] experiment, common buckwheat (*Fagopyrum esculentum* Moench. variety ‘Darja’), was grown in outdoor experiments under a reduced, ambient, and increased UV-B level. It was shown that, during plant development, the UV-B-absorbing compounds were increased by UV-B radiation. In addition, an analysis of the influence of ambient UV radiation showed the increased synthesis of protective substances in hybrid buckwheat [31].

It was established that there are differences in the reactions of plants in the concentration of rutin after the short-term exposure of buckwheat plants to UV-B radiation. The exposure of cv. Hruszowska is associated with a lower concentration of rutin. The high resistance of cv. Red Corolla to UV radiation is possibly connected to the high contents of rutin and total anthocyanins, in comparison with other cultivars. Short-term UV-B exposure increased the content of anthocyanins and inhibited the elongation growth of hypocotyls [32,33].

The atmospheric ozone layer, which enables favorable UV levels at the Earth’s surface, will presumably recover to the long-term mean recorded from 1964 to 1980 until 2050 [34]. Buckwheat is a plant that originated in high-altitude habitats with elevated UV levels; thus, it is probably a suitable crop for culturing under enhanced UV-B radiation [16].

UV-B radiation may have adverse impact on plants by damaging membranes, proteins, and DNA [35]. Elevated UV-B radiation more strongly lowered the chlorophyll level and effective photochemical efficiency of Photosystem II of Tartary buckwheat than water limitation.

The decrease in the biomass production in common buckwheat when subjected to water limitation and enhanced UV-B radiation was significant, while it was only limited in Tartary buckwheat [36]. This might be a consequence of the high concentration of rutin in Tartary buckwheat [37]. UV-radiation absorption is not the only function of UV-absorbing substances. There is rutin in the grain of buckwheat, and especially in the outer layers and in the cotyledons. In addition to rutin, there are also enzymes in the grains of Tartary buckwheat, which allow the rutin to be transformed into quercetin. Both molecules, rutin and quercetin, are quite similar, except that rutin has two sugar molecules attached to the quercetin-part of the molecule. The enzyme rutinase allows the secession of the sugar part and changes rutin into a quercetin. This enzyme is located in the other tissue of the buckwheat grain as rutin, and so they are spatially separated. Thus, in the intact grain, rutin is protected from decomposition. Ripe buckwheat grains are not very attractive to animals due to the hard husk and three sharp edges. An animal, such as a deer, starts to graze on unripe buckwheat grain, grinds it between the teeth, and mixes it with saliva. Rutinase molecules are mixed with rutin by chewing in a humid environment while grazing, and when conditions are suitable for breaking down rutin molecules and forming quercetin. Thus, the bitter quercetin begins to appear. Because of the bitter taste, animals are discouraged to graze on Tartary buckwheat. Tartary buckwheat has significantly more rutin in the grain than common buckwheat. Deer, when the plants of both common and Tartary buckwheat are available, prefer to graze on the young upper parts of common buckwheat and avoid Tartary buckwheat [38,39,40]. 

Secondary metabolites of buckwheat with benzene rings also protect the plants in other ways. They protect buckwheat grain from fungal attacks. This is particularly important when it comes to fungi that form mycotoxins [41,42]. Among flavonoids, the potential effect of rutin, quercetin, and kaempferol against mycotoxin production in *Aspergillus* was observed. Chitarrini et al. [43] studied the antimycotoxin impact of rutin and quercetin in vivo and suggested that Tartary buckwheat, rich in antioxidants, is resistant to *A. flavus* contamination. Thus, Tartary buckwheat is more resistant to infection with fungi than common buckwheat.

Buckwheat and its relatives, such as rhubarb (*Rheum rhabarbarum* L.), contain calcium oxalate (CaOx) crystals that further protect plants from the Sun’s rays and play a role in protecting them from aluminum toxicity [44]. Peng et al. [45] found differences in the resistance to aluminum, and oxalate formation between the genotypes of common buckwheat. Breeding to reduce the oxalate content could increase the plants’ sensitivity to aluminum or solar radiation.

It has also been shown that, in buckwheat, CaOx druse crystals can provide protection against solar radiation. For example, the density of CaOx druses in Tartary buckwheat positively correlated with the reflectance in the blue, green, yellow, and UVB regions of the spectrum [46].

In plants, the growth is decreased with the intensity of UV irradiation. UV treatment effectively activates the expressions of secondary-metabolite biosynthetic genes, and also in *Linum*, canola, soybeans, and other plants, depending on the UV-irradiation intensity and time of impact [47,48,49]. UV-B exposure may increase the nutritional and nutraceutical quality of crop plants [47]. Melatonin was reported as an enhancer of the synthesis of flavonoids under exposure to UV-B radiation by promoting the gene expressions for enzymes involved to flavonoid synthesis [49].

Buckwheat is known to be a suitable crop for food biofortification with selenium, and it can accumulate a substantial amount of Se. Se is reported to interfere with the reaction of buckwheat plants in cases of UV-B radiation and drought conditions [16,31,50,51]. The addition of Se mitigated the negative effect of the UV-B radiation on the effective photochemical efficiency of Photosystem II in Tartary and common buckwheat. Se treatment also ameliorated the stunting effect caused by UV-B radiation, as well as the lowering of the biomass in common buckwheat [50]. The combination of the Se treatment and ambient UV radiation resulted in lower yields of hybrid buckwheat compared with untreated plants. However, under these conditions, the buckwheat plants established good protection against the different environmental stresses due to the increased concentration of phenolic compounds [31]. After foliar treatment, buckwheat can be a source of Se for the human diet [31,52,53].

Notably, patients with severe COVID-19 infection have vitamin D and Se deficiencies [54]. Indeed, as Se appears to enhance the cytotoxic effector cells, Se deficiency is a possible risk factor for COVID-19 mortality [54]. Se-enriched Tartary buckwheat, which is also rich in flavonoids and other nutrients, could be a suitable preventive diet for people at risk of becoming infected with severe COVID-19 [55].

## 3. Human-Health-Related Effects of Buckwheat

Buckwheat has high nutritional value, including a high total vitamin B content [56] and very high levels of antioxidants, such as rutin [16,57]. Rutin is a flavonoid known for its ability to strengthen blood vessels, aiding the effect of vitamin C, and providing many other potential health benefits, such as reductions in cholesterol levels and blood clots [58,59,60]. Foods made from the grain of Tartary buckwheat have shown preventive effects against several chronic diseases, including obesity, cardiovascular diseases, gallstone formation, and hypertension. The effects are mainly attributed to the phenolic substances, resistant starch, the slowly digestible proteins in the grain, and especially to the interactions among these constituents [53]. Polyphenols have an impact on protein digestibility after hydrothermal treatment. Their interaction reduces the digestion of proteins through the small and large intestines. Microbial processes in the colon enhance the digestibility of the grain proteins and starch, which are otherwise blocked by polyphenols in hydrothermally processed buckwheat. Yu et al. [61] compared the rutin and quercetin contents across 44 Tartary-buckwheat grain and sprout samples from China, Nepal, Bhutan, India, Japan, Pakistan, and Slovenia. They were very variable for these different origins [53]. The samples from Nepal had the highest concentrations of rutin in the grain (13.3 g/kg) and sprouts (54.4 g/kg). For quercetin, the sprouts contained 10–90 fold that found in the grain [61]. Tartary-buckwheat sprouts have great potential for products rich in flavonoids.

A comparison of Tartary buckwheat and common buckwheat showed a big difference in the metabolic profiles between the two buckwheat species. It was found that 61 flavonoids and 94 nonflavonoid secondary metabolites showed higher contents (at least two fold) in Tartary buckwheat than in common buckwheat. It is suggested that Tartary- and common-buckwheat grains are rich in secondary metabolites that are beneficial to human health, among them, nonflavonoid metabolites also contributed to Tartary buckwheat’s higher health-promoting value in comparison with common buckwheat [62,63]. Zhang et al. [5] reported the sequencing and assembly of Tartary buckwheat. 

The milling of the grain of Tartary buckwheat and mixing the flour with water results in the formation of quercetin as a degradation product of rutin, degraded by rutinosidase [12,64,65,66,67,68,69]. Ingested quercetin can cross the blood–brain barrier and accumulate in the brain tissue [70]. Indeed, important bioactivities have been established for quercetin and its derivatives not just in blood vessels, muscles, and the gastrointestinal system, but also in the brain. Quercetin and its glycosides exert antioxidant and anti-inflammatory actions through multiple mechanisms, including the activation of the Nrf2/ARE pathway and the downregulation of the nuclear factor kappa B pathway [71,72]. Quercetin and other phenolics have been isolated from the stool samples of people who had eaten food rich in phenolic substances [73,74,75]. The presence of phenolic substances in the colon could modulate the microbiota composition in the stools [76].

In Tartary buckwheat, quercetin complexation with starch molecules impacts the in vitro digestibility of the starch and the appearance of resistant starch, thus altering the physicochemical properties of the Tartary-buckwheat starch [77]. The effects of this quercetin–polyphenol complexation indicate that food products based on Tartary buckwheat will show lower digestibility. Indeed, the quercetin in Tartary buckwheat can reduce the body weight, serum triacylglycerols, and low-density lipoprotein. In rats, a diet with 0.1% quercetin was shown to have significant effects towards lowering the low-density-lipoprotein concentrations in serum, with no such effects on the high-density lipoprotein. Tartary buckwheat has also been shown to prevent increases in body weight and fat deposition during high-fat intake in rats, although this was reported to protect against hepatic stenosis [78]. A buckwheat diet can also reduce insulin and ameliorate glucose intolerance in humans [79].

The slow digestibility of Tartary-buckwheat starch appeared due to the impact of phenolic substances on the starch [80,81]. In in vivo experiments, mice showed reduced postprandial glycemic responses. Similar results were obtained in common buckwheat [79].

In a rat model, Suzuki et al. [65] investigated the possibilities for toxicity of rutin-rich dough made from Tartary buckwheat by acute and subacute toxicity studies (10,000 and 5000 mg flour per kg body weight, respectively). The concentration of rutin in the Tartary-buckwheat material was 1570 mg/100 g. In the experiment, no toxic symptoms or unusual symptoms were observed. The body weight was not significantly different among the groups of animals. The authors concluded, based on the results, that buckwheat at a given dose is at a noneffect level. The results of Suzuki et al. [82] and Vogrinčič et al. [83] also suggested that the studied buckwheat-grain material was not genotoxic.

Among buckwheat secondary metabolites, fagopyrin appears to be a health threat when the green parts of the plants are consumed. On the one hand, the ingestion of buckwheat grain and related grain food products has been shown to be safe regarding the low concentrations of fagopyrin [28,84,85,86]. On the other hand, fagopyrin may have a protecting effect against *Phytophthora* [87].

## 4. Conclusions

At high altitudes, the plants of common and Tartary buckwheat survived during evolution by gradually accumulating genes for the synthesis of substances, which allowed the plants to survive and reproduce in the less friendly environment. Both the diversity and richness of Tartary-buckwheat substances protect the plants from ultraviolet radiation, diseases, and grazing. These substances are mainly phenols (i.e., substances with aromatic rings made of six carbon atoms, with double ties and the carbon-atom-bound groups OH or others. Often other molecules, such as sugars and proteins, are attached. Among the particularly interesting metabolites of buckwheat are rutin, quercetin, and fagopyrin.

The flavonoids rutin and quercetin in buckwheat are mainly allocated in the leaves and flowers. A small concentration of rutin was found in the grain of common buckwheat, while up to 1.4% rutin was found in the grain of Tartary buckwheat.

Many of these substances are also important in nutrition to preserve human health. Rutin is a flavonoid known for its ability to strengthen blood vessels, aiding the effect of vitamin C, and providing many other potential health benefits, such as reductions in cholesterol levels and blood clots.

In Tartary buckwheat, quercetin complexation with starch molecules impacts the in vitro digestibility of the starch and the appearance of resistant starch, thus altering the physicochemical properties of the starch. Polyphenols have an impact on protein digestibility after hydrothermal treatment. Their interaction reduces the digestion of proteins through the small and large intestines. Microbial processes in the colon enhance the digestibility of the grain proteins and starch, which are otherwise blocked by phenolic substances.

Plant products obtained from the grains of common and Tartary buckwheat have shown preventive impacts against cardiovascular diseases. These properties are mainly attributed to the phenolic substances and the molecular interaction of starch, proteins, and phenolic substances, including the flavonoids rutin and quercetin.

## Figures and Tables

**Table 1:** Contents of total flavonoids and fagopyrins in parts of buckwheat in mg RE (rutin equivalents)/g DW (dry weight) (adapted from Zielinska et al., 2012) [27], and in mg HE (hypericin equivalents)/g DW (adapted from Kim and Hwang, 2020) [28]; /: no data.

Part of Plant	Common Buckwheat		Tartary Buckwheat	
	Flavonoids (mg RE/g DW)	Fagopyrins (mg HE/g DW)	Flavonoids (mg RE/g DW)	Fagopyrins (mg HE/g DW)
Stems	/	1.20	/	2.00
Leaves	82	1.03	78	0.56
Flowers	204	2.25	145	8.06
Ripe seeds	6	/	20	/

## Data Availability

The data presented in this study are available upon request from the first and corresponding author.
